# Expression patterns of transcribed human endogenous retrovirus HERV-K(HML-2) loci in human tissues and the need for a HERV Transcriptome Project

**DOI:** 10.1186/1471-2164-9-354

**Published:** 2008-07-29

**Authors:** Aline Flockerzi, Alessia Ruggieri, Oliver Frank, Marlies Sauter, Esther Maldener, Bernd Kopper, Bernd Wullich, Wolfgang Seifarth, Nikolaus Müller-Lantzsch, Christine Leib-Mösch, Eckart Meese, Jens Mayer

**Affiliations:** 1 Department of Human Genetics, Medical Faculty, University of Saarland, Homburg, Germany; 2 Medical Clinic III, Medical Faculty Mannheim, Ruprecht-Karls-University Heidelberg, Mannheim, Germany; 3 Institute of Virology, Medical Faculty, University of Saarland, Homburg, Germany; 4 Clinic of Urology and Pediatric Urology, Medical Faculty, University of Saarland, Homburg, Germany; 5 Helmholtz Zentrum München, German Research Center for Environmental Health, Institute of Virology, Neuherberg, Germany; 6 Institute for Molecular Cell Biology, Medical Faculty, University of Saarland, Homburg, Germany; 7 Clinic of Urology, Erlangen University Hospital, Erlangen, Germany

## Abstract

**Background:**

A significant proportion of the human genome is comprised of human endogenous retroviruses (HERVs). HERV transcripts are found in every human tissue. Expression of proviruses of the HERV-K(HML-2) family has been associated with development of human tumors, in particular germ cell tumors (GCT). Very little is known about transcriptional activity of individual HML-2 loci in human tissues, though.

**Results:**

By employing private nucleotide differences between loci, we assigned ~1500 HML-2 cDNAs to individual HML-2 loci, identifying, in total, 23 transcriptionally active HML-2 proviruses. Several loci are active in various human tissue types. Transcription levels of some HML-2 loci appear higher than those of other loci. Several HML-2 Rec-encoding loci are expressed in GCT and non-GCT tissues. A provirus on chromosome 22q11.21 appears strongly upregulated in pathologic GCT tissues and may explain high HML-2 Gag protein levels in GCTs. Presence of Gag and Env antibodies in GCT patients is not correlated with activation of individual loci. HML-2 proviruses previously reported capable of forming an infectious HML-2 variant are transcriptionally active in germ cell tissue. Our study furthermore shows that Expressed Sequence Tag (EST) data are insufficient to describe transcriptional activity of HML-2 and other HERV loci in tissues of interest.

**Conclusion:**

Our, to date, largest-scale study reveals in greater detail expression patterns of individual HML-2 loci in human tissues of clinical interest. Moreover, large-scale, specialized studies are indicated to better comprehend transcriptional activity and regulation of HERVs. We thus emphasize the need for a specialized HERV Transcriptome Project.

## Background

The human genome harbors a significant amount of sequences that stem from retroviral infections of the germ line in evolutionarily ancient times, so-called human endogenous retroviruses (HERVs). Repeated (re)infection by different exogenous retroviruses, and intracellular amplification of endogenous retroviral sequences, or composite elements with retroviral portions, resulted in about 8% of the human genome having a retroviral origin. A great number of distinct HERV families have been defined that each stem from germ line infections of distinct exogenous retroviruses. Many of the integrated retroviruses (proviruses) became defective due to accumulation of nonsense mutations, large internal deletions, or reduction to so-called solitary LTRs after homologous recombination within a provirus [for reviews, see [1-4]].

Since proviruses carry their own transcriptional promoters and regulators within the Long Terminal Repeats (LTRs), HERV sequences are able to initiate transcription of the proviral *gag*, *pro*, *pol *and *env *genes, but also to initiate transcription of neighboring cellular genes. Splice signals within HERVs can also result in variant transcripts of cellular genes. Various examples have been well documented where HERV sequences influence the transcription of cellular genes or alter the structure of cellular transcripts [for instance, see references [5-11]]. In accord, HERV sequences display characteristic distributions relative to genes [12]. It appears that HERV sequences are much more likely to loose coding-capacity due to nonsense mutations than they loose their promoter activity. Therefore, many HERV promoter sequences still display transcriptional activity after millions of years in the genome. In fact, recent studies demonstrated that there is virtually no human tissue that lacks HERV transcripts, and transcripts from several HERV families are usually found in every investigated human tissue [13,14].

The regulation of transcriptionally active HERV sequences is, as of yet, little understood. While chromatin status probably contributes to their regulation, CpG methylation status of HERV promoter and regulatory regions appears as a crucial factor for activity versus inactivity [15-17]. However, relatively little is known about transcription factors actually regulating transcriptional activity of individual HERV loci [11].

Expression of HERV sequences has been proposed to be involved in the etiology of various human diseases. However, no direct connection could be established in most cases so far [18,19]. An involvement in human disease has been shown particularly for the human endogenous retrovirus family HERV-K(HML-2), in short, HML-2, that is exceptional for various reasons. While there are a number of evolutionarily old HML-2 loci in the human genome [20] evolutionarily young HML-2 loci have been proposed to have formed in the human lineage by reinfection rather than an intracellular copying mechanism, raising the possibility that an infectious HML-2 variant is present in the human population until today [21,22]. An infectious and replication-competent HML-2 variant was recently engineered from a consensus sequence of evolutionarily young HML-2 loci [23,24]. Recent HML-2 activity also resulted in a number of HML-2 loci that are polymorphic in the human population due to incomplete fixation [25-30].

It is known that HML-2 sequences are drastically upregulated in germ cell tumors (GCT), the most frequent tumor among young men. The precursor lesion of GCT, the carcinoma *in situ*, already displays strong HML-2 expression [31]. HML-2 is also exceptional because of its coding capacity for Gag, Pro, Pol and Env proteins in that several HML-2 loci in the human genome still encode those proteins [for reviews, see [1,32]]. Gag protein is readily detectable in GCT tissue and GCT patients display high antibody titers against HML-2 Gag and Env proteins at the time of tumor detection [33,34]. Furthermore, some HML-2 loci encode a functional homologue of the HIV_Rev _protein, named Rec, that interacts with PLZF (a protein involved in spermatogenesis in the mouse) and that results in disturbed spermatogenesis in Rec-transgenic mice [35,36]. Another HML-2 protein, Np9, is encoded by some mutated HML-2 loci that lack a 292 bp sequence at the *pol*/*env *boundary, so-called type 1 proviruses, as opposed to type 2 proviruses. Np9 may be involved in GCT development as well [37]. Expression of HML-2 RNA and proteins has also been investigated in more detail in melanoma [38-40]. The role of HML-2 in both GCT and melanoma remains to be clarified.

While upregulation of HML-2 in GCT is well documented, little is known about HML-2 loci actually being activated in the course of GCT development. Do higher transcript levels in GCT result from drastic upregulation of only a few HML-2 loci or from upregulation of a greater number of loci? What HML-2 loci are expressed in GCT tissue and in corresponding normal testicular tissue? More generally, what HML-2 loci are transcriptionally active in various human tissues? Especially the latter question is legitimate for all other HERV families as well. In fact, there is very restricted or no information at all on what specific HERV loci contribute to the cellular RNA pool in various tissues and how expression patterns of individual HERV loci vary between, for instance, healthy and diseased conditions. Recent analysis employed molecular genetic and bioinformatic means to identify HERV transcripts. First, HERV-derived cDNAs have been generated and assigned to individual HERV loci in smaller scale studies [41-45], and more recently, for ERVs in the mouse [46]. Second, recent analysis employed sequence data deposited in the human section of the Expressed Sequence Tag database dbEST [47] in order to identify transcriptionally active HML-2 and other HERV loci [48,49]. A number of active loci have been identified by doing so.

However, we will demonstrate here that sequence data in dbEST are insufficient for a detailed description of HERV expression patterns in various cell types and cellular conditions. Yet, given their quantity, a more comprehensive investigation of the transcriptional activity of HERV loci appears fundamental for better understanding the transcriptional activity of the human genome as a whole. Recent results from the pilot phase of the Encyclopedia of DNA Elements (ENCODE) Project provided both fascinating and provocative insights into the transcriptional activity of the human genome, especially regarding pervasive transcription of the human genome [50-52]. Considering their inherent promoter activity and their ubiquitous expression, HERV sequences may be responsible for a significant number of transcripts.

In the present study, we communicate results from the largest-scale identification of transcriptionally active HML-2 loci so far, and their expression patterns in human tissues of interest. We compare our results to previously published work by others and us. We conclude that a specialized, potentially collaborative HERV cDNA sequencing project, a *HERV Transcriptome Project*, is needed to comprehensively describe transcriptional activities of HERV loci in human normal and diseased tissues.

## Results

### Generation of HML-2 cDNA sequences

We analyzed, in total, 49 samples derived from tumor and normal tissues that were of higher interest because of various previous findings. In detail, we analyzed 15 samples from testicular tissue including 10 different seminoma samples and 5 samples from normal or non-malignant testis. We analyzed 15 brain samples including 5 samples from normal brains, 5 samples from patients with a bipolar disorder and 5 samples from patients with schizophrenia. We analyzed 9 brain tumors of different stages of malignancy (common type meningiomas, atypical meningioma, meningioma grades II and III, glioblastoma multiforme). We also analyzed the following samples: a mammary carcinoma and a normal mammary tissue, a lung carcinoma and a normal lung tissue, the germ cell tumor-derived cell line Tera-1 and the mammary carcinoma-derived cell line T47D.

**Figure 1:**
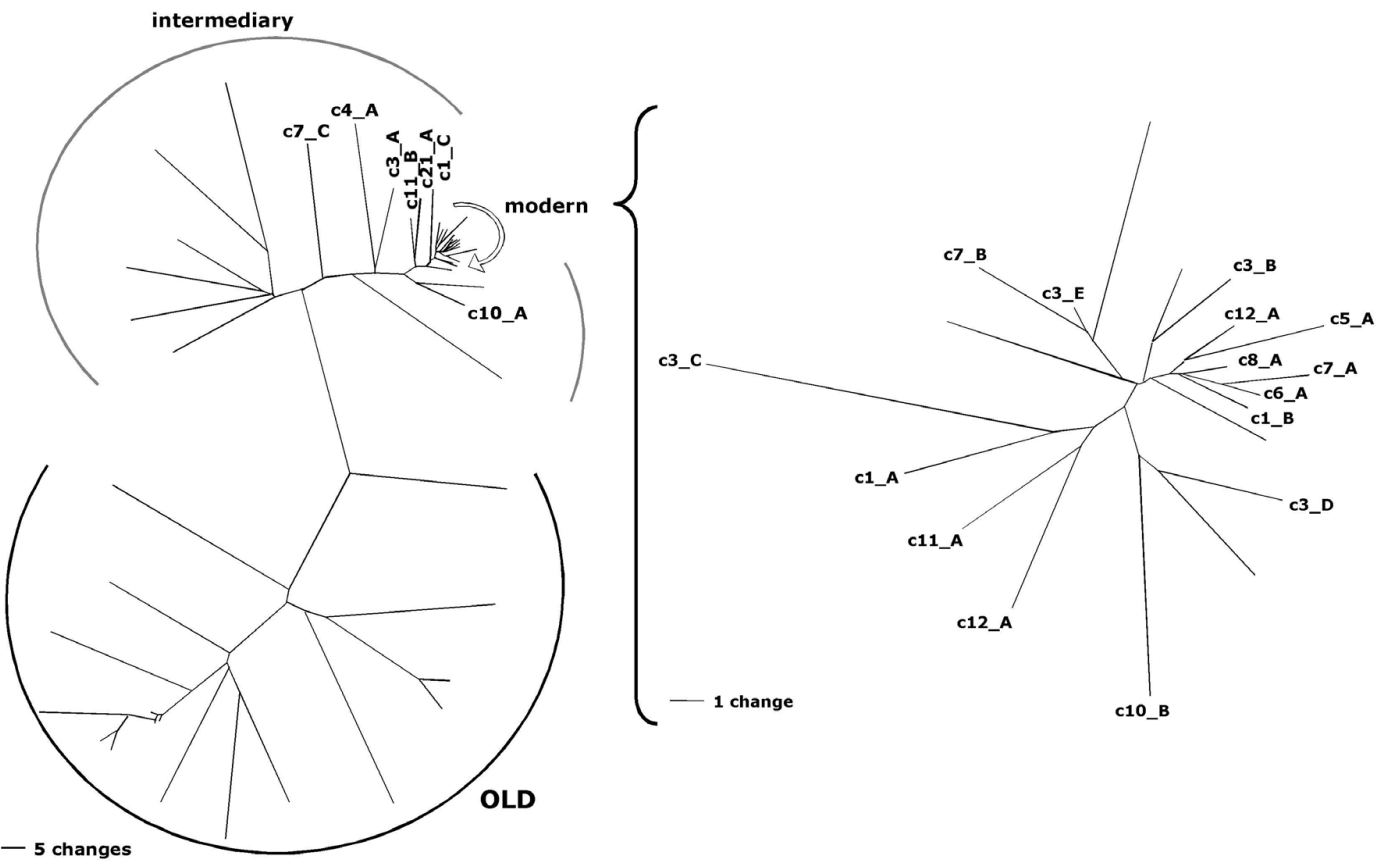
**Neighbor joining (NJ) trees of HERV-K(HML-2) sequences in the proviral reference sequence dataset for *gag*-derived cDNAs**. The NJ-trees depict absolute numbers of nucleotide differences between the various sequences, excluding indels for pairwise comparisons, thus demonstrating private nucleotide differences between HML-2 loci. The tree on the right depicts a subset of sequences, labeled "modern" in the tree on the left, at higher resolution. Only HML-2 loci having the amplified *gag *region were included in the tree. The investigated *gag *region also includes a 96 bp region that distinguishes evolutionarily modern HERV-K(HML-2) from evolutionarily old (HERV-K(OLD)) proviruses. An intermediary group between modern and old proviruses is evident. For better resolution, a separate NJ tree for the modern HML-2 proviruses is shown at the right. For the sake of simplicity, only branches of proviruses identified as transcriptionally active in this study are indicated in the tree, other branches are unlabelled. Scale bars represent 5 nt differences in the left tree and 1 nt difference in the right tree.

We performed RT-PCR on RNA isolated from each specimen employing HML-2-specific PCR primer pairs that were located within the central region of the HML-2 *gag *gene and the 3' region of the HML-2 *env *gene, generating PCR products of about 650 bp and 500 bp, respectively. In total, forty-seven samples were subjected to a *gag*-specific RT-PCR and 30 samples were subjected to an *env*-specific RT-PCR. Following RT-PCR, products were cloned and individual cDNAs were sequenced and assigned to specific HML-2 proviruses in the human genome based on private (one or several nucleotides that are characteristic for an HML-2 locus) nucleotide differences between individual loci depicted in Fig. 1. An in-house Bio-Python script, LOCUS-ASSIGNER, was employed for that purpose. The script is available from the authors on request.

We generated, in total, 1587 cDNA sequences; 975 for *gag *and 612 for *env*. Among those, 327 cDNAs were generated from brain tumor samples, 374 cDNAs were generated from normal brain, and brains with bipolar and schizophrenic condition, and 666 cDNAs were generated from testicular samples. We generated up to 49 cDNAs for each tissue specimen, with an average of 30 (SD 13.7) cDNAs per specimen. We restricted interpretation of results for specimens with low numbers of generated cDNA sequences, such as for lung tissue and T47D. Nevertheless, those data have been included in Additional File 1 for the sake of completeness. Results for the Tera-1 cell line and retroviral particles produced by Tera-1 cells will be described in more detail elsewhere (Ruprecht et al., manuscript submitted).

Based on our recently published data [53], we excluded 5.5% of cDNA sequences (6.9% for *gag *and 3.4% for *env*) from the analysis because they very likely represented *ex vivo *recombined cDNAs from transcripts from different HML-2 proviral loci, evidenced by a significantly higher number of mismatches for the best cDNA-provirus match.

We thus analyzed 1499 cDNA sequences, that is, those cDNA sequences were assigned to individual HML-2 proviral loci, identifying the particular proviral locus as transcriptionally active in the regarded tissues. The below given relative cloning frequencies indicate for each HML-2 locus how often *gag *or *env *individual cDNA sequences generated from a particular tissue sample could be assigned to a particular locus relative to the total number of assignable *gag *or *env *cDNAs for that tissue sample. For example, a relative cloning frequency of 20% for locus c1_A means that 8 out of 40 cDNA clones could be assigned to c1_A for a given tissue sample. When appropriate, results from several tissue samples were combined. Absolute numbers of transcriptionally active HML-2 loci indicate how many different loci were represented by the total number of cDNAs for a given tissue sample.

### Transcriptionally active HML-2 proviruses

Overall, our analysis detected 23 transcriptionally active HML-2 proviruses in the human genome. Twenty-one proviruses were detected by *gag*-derived cDNAs and 14 proviruses were detected by *env*-derived cDNAs. Nine proviruses were detected exclusively by *gag *cDNAs and 2 proviruses were detected exclusively by *env *cDNAs. Transcriptionally active HML-2 proviruses were located on 12 different human chromosomes, with 5 proviruses being located on human chromosome 3 (Table 1). Results for all investigated tissue specimens have been summarized in Additional file 1.

**Table 1:** Features of transcriptionally active HERV-K(HML-2) loci identified in this study^1^.

	**Transcripts**		**ORF**														
			
	**gag**	**env**	**gag**	**env**	**type 1/2**	**5' LTR**	**age**	**band**	**location amplicon in genome**			**Hughes '04 polymorphic**	**Stauffer '04 ESTs**	**Mayer '04 Rec ORFs**	**Lavie '05**	**Buzdin '06 cDNAs**	**Dewannieux '06**
**c1_A**	+	+			1	+	h	1p31.1	1	75616984	75617620	1p31				1p31.1	
**c1_B**	+	+	(+)		1	+	h	1q22	1	153869999	153870635		1q22*			1q23.1#	
**c1_C**	+				1	+	h	1q23.3	1	158928816	158929450						
**c3_A**	+	+			1	+	h	3p25.3	3	9869025	9869661						
**c3_B**	+	+	+		1	+	h	3q13.2	3	114232719	114233355		3q13.2*			3q13.2	
**c3_C**	+	+			2	+	h	3q21.2	3	127093452	127094088			3q21.2		3q21.2	
**c3_D**	+				2	-	h	3q24	3	149766793	149767429						
**c3_E**	+		+		1	+	h	3q27.2	3	186769952	186770588					3q27.2	
**c4_A**	+				1	+	och	4q32.3	4	166137900	166138546					4q32.1	
**c5_A**	+	+	+		1	+	h	5q33.3	5	156024213	156024849					5q33.3	
**c6_A**	+	+	+	+	2	+	h	6q14.1	6	78490549	78491185	K109		6q14.1	6q14.1	6q14.1	K109
**c7_A**	+	+	+	+	2	+	h	7p22.1	7	4604296	4604932	K108		7p22.1	7p22.1	7p22.1	K108
**c7_B**	+					-	h	7q22.1	7	104179212	104179848					7q22.2#	
**c7_C**	+				OLD	-	roch	7q34	7	141100988	141101724						
**c8_A**	+				2	+	h	8p23.1	8	7350016	7350652	K115				8p23	K115
**c10_A**	+	+			2	+	ch	10p14	10	6913351	6913990		10p14	10p14	10p14		
**c10_B**	+				2	-	h	10q24.2	10	101576412	101577048			10q24.2		10q24.2	
**c11_A**	+	+		+	2	+	h	11q22.1	11	101072629	101073265	11q22		11q22.1	11q22.1	11q22.1	
**c11_B**	+				1	+	ch	11q23.3	11	118103837	118104468						
**c12_A**		+	+	+	2	+	h	12q14.1	12	57014704	57015340	12q14	12q14	12q14.1	12q14.1	12q14.1	
**c19_A**		+				-	h	19p13.3	19	337322	340413						
**c21_A**	+	+			2	+	h	21q21.1	21	18861736	18862372					21q21.1	
**c22_A**	+	+	+		1	+	h	22q11.21	22	17307812	17308448					22q11.21	

^1 ^Provirus designations are given in the first column. Each provirus was identified (indicated by "+") by *gag*- and/or *env*-derived cDNAs. Presence of ORFs for Gag and Env proteins was deduced from proviral sequences as given in the March 2006 version of the human genome sequence at the Human Genome Browser. The Gag ORF in provirus c1_B harbors a premature stop in the human genome reference sequence but a full-length ORF allele appears to be the more frequent allele in Caucasians (own unpublished data). Provirus type 1 or 2, based on a 292 bp sequence within the *pol*-*env *boundary (if the proviral region is present), presence of a 5'LTR. "age" indicates presence of orthologous HML-2 loci in rhesus (r), orang-utan (o), chimpanzee (c) and/or human (h) genomes, thus indicating evolutionary ages of loci. The chromosomal band in which the provirus is located, and the nucleotide position of the RT-PCR amplicon amplified during cDNA generation are given in the following columns. Locus c7_A is an HERV-K(OLD) provirus. For c19_A the position of the *env *amplicon is given. We compared our results with other recent publications (see text for details and references). Hughes '04 denotes proviruses that were identified as polymorphic in the human population. Stauffer '04 denotes proviruses previously identified as transcriptionally active by analysis of EST data. "*" indicates that transcriptionally activity of those proviruses was ambiguous based on EST sequence data. Mayer '04 denotes proviruses with ORFs for Rec protein. Lavie '05 denotes proviral 5'LTRs previously examined for promoter activity. Note that the 5'LTR of provirus c10_A displayed very low transcriptional activity in the previous study when in Tera-1 cells in transient reporter assays. Buzdin '06 denotes transcribed proviruses identified by means of GREM (see text). "#" indicates that a different chromosomal band was given for that locus, but sequence comparisons show that corresponding loci are identical with the loci identified in our study. Dewannieux '06 denotes proviruses reported as capable of creating an infectious HML-2 variant by recombination. See the text for above mentioned references. Note that provirus designations in other publications are different. Great care has been taken to relate provirus designations mentioned in other studies with proviruses mentioned in this study.

As for the structure of transcriptionally active HML-2 proviruses, most of them are intact in that they consist of *gag*, *pro*, *pol *and *env *sequences and flanking 5' and 3' LTRs (see Additional file 2). Based on the presence or absence of a 292 bp sequence within the *pol*-*env *boundary, both type 2 (n = 10) and type 1 (n = 10), respectively, HML-2 proviruses are transcriptionally active. The structure of the less intact loci is as follows (see Table 1 for details on provirus designations). Provirus c1_A lacks a *pol *region (~nt 3500–6300 with respect to the HERV-K(HML-2.HOM) sequence [54]). Proviruses c3_A and c4_A lack a *pol *region (~nt 3700–5900). Provirus c10_B lacks the 5'LTR and an approx. 1.3 kb long *env *region. Provirus c19_A consists of only an *env*-3'LTR portion (~nt 7000-end). Provirus c3_D lacks the 3' half of *pol*, the *env *gene and the 3'LTR. Provirus c7_B and c7_C consist of *gag*-*pro*-*pol *sequence (~nt 1000–4900), with provirus c7_C being flanked by an LTR in opposite orientation. Lack of proviral regions in some proviruses also explains the above-mentioned exclusive detection of some proviruses as either *gag *or *env*-derived cDNAs. As for the evolutionary ages of active HML-2 loci, comparative genomics tracks at the UCSC Genome Browser [55] show that 18 out of 23 active HML-2 loci are human-specific, thus less than approx. 6 million years old. Three loci were specific for human and chimpanzee, one locus was present in orang-utan, chimpanzee and human. Notably, provirus c7_C belongs to the evolutionary precursor of "modern" HERV-K(HML-2.HOM) sequences, the so-called HERV-K(OLD) subfamily, as indicated by a 96 bp insertion within *gag *[20] and its presence in rhesus, orang-utan, chimpanzee and human.

Based on the human genome reference sequence as given in the March 2006 version at the UCSC Human Genome Browser [55], several of the transcriptionally active HML-2 proviruses harbor open reading frames (ORFs) for HML-2 genes previously reported to be associated with GCT and other tumors. Specifically, 7 transcribed proviruses harbor *gag *ORFs and 4 proviruses harbor *env *ORFs. A previously published study [42] reported that 7 transcribed proviruses harbor an ORF for the HML-2 Rec protein (see below). Notably, three proviruses (c6_A, c7_A and c12_A) harbor ORFs for Gag, Env and Rec (Table 1). According to gene annotations in the Human Genome Browser, 6 transcribed proviruses are located within gene introns: c1_A: SLC44A5; c1_C: CD48; c5_A: SGCD; c7_B: LHFPL3; c8_A: DEFB107; c10_B: ABCC2. All proviruses were located in antisense orientation with respect to the direction of the genes' transcription.

### HML-2 provirus expression in brain tissues

**Figure 2:**
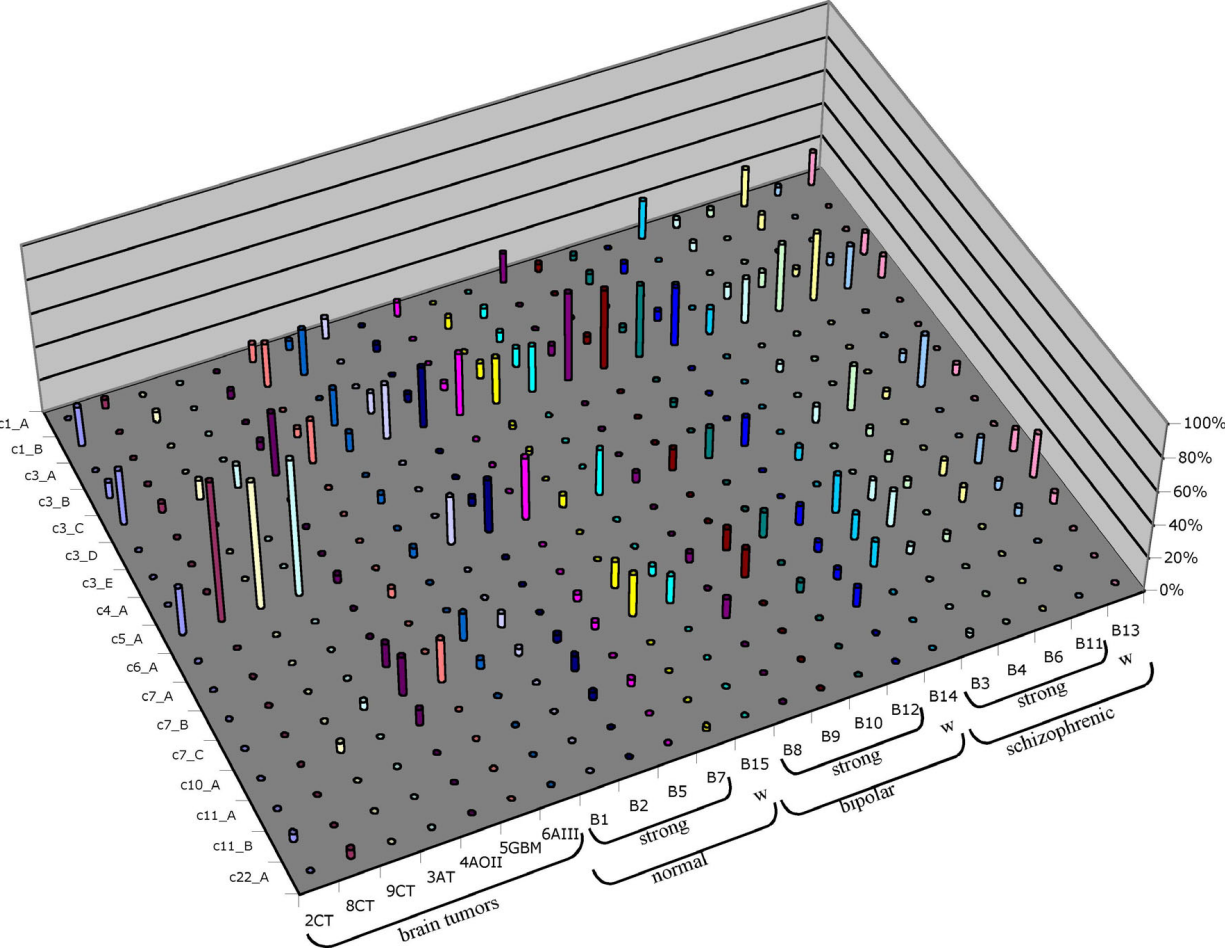
**Transcriptional activity of HERV-K(HML-2) proviruses in different brain tissues**. For each provirus, the relative cloning frequency of *gag*-derived cDNAs per tissue specimen that were assignable to the particular provirus are given. Only proviruses for which transcripts have been found as cDNA are included. Brain tissue specimens have been grouped in those derived from various types of brain tumors (CT: common type meningiomas; AT: atypical meningioma; AOII: meningioma grade II; GBM: glioblastoma multiforme; AIII: atypical meningioma grade III) and in those from normal, bipolar and schizophrenic patients. Specimens with previously identified stronger (strong) and weaker (w) HML-2-specific signal intensities in microarray experiments (see text) are indicated. See Additional file 1 for more details.

We next analyzed the transcriptional activity of individual HML-2 proviruses in the various brain tissue specimens. A total of 21 HML-2 proviruses were found transcribed in 23 different brain tissue specimens. On average, 7.1 (SD 1.9) proviruses were found expressed in each sample. There was no obvious difference between specimens regarding the number of transcribed proviruses or combinations of transcribed proviruses. Brain tumor samples displayed, on average, 6.5 (SD 2.9) transcribed proviruses. Samples from bipolar and schizophrenic conditions and normal brain displayed 6.8 (SD 1.1), 8 (SD 1.2) and 7.4 (SD 1.1), respectively, transcribed proviruses. Transcripts from about 13 proviruses were detected only rarely, that is, cDNAs from those loci were identified sporadically. For instance, proviruses c3_A and c3_D were each represented by only one out of 654 brain-derived *gag *and *env *cDNAs. Only 4 out of 654 cDNAs could be assigned to provirus c22_A. On the other side, cDNAs from about 5 proviruses (c3_B, c3_C, c5_A, c7_B, c7_C) were found in almost every brain sample. For instance, transcripts from proviruses c3_C and c5_A were found in 22/22 and 20/22, respectively, brain samples with higher cDNA clone numbers (Fig. 2).

A previous study revealed differences in HML-2 transcript levels in human brain samples by means of a *pol*-based HERV-specific microarray that included four slightly different HML-2-specific oligonucleotide probes [56]. Specifically, for some of those oligonucleotide probes several brain samples displayed significantly stronger HML-2 signal intensities in microarray experiments than other samples, suggesting differential expression of (a subgroup of) HML-2 loci. For the present study cDNA previously generated from brain samples from healthy donors as well as bipolar and schizophrenic conditions were selected based on strong and weak HML-2 signal intensities in that 1 out of 5 samples for each condition had originally shown significantly weaker signal intensities [56]. In total, 16 HML-2 proviruses were found expressed in those brain samples. As above, proviruses c3_C and c5_A were expressed at higher levels than others while transcripts from proviruses c3_A, c3_D, c7_A and c22_A were found transcribed only rarely. For the transcriptionally active HML-2 loci detectable in our study there was no obvious correlation between expression of specific proviruses and previously observed signal intensities (Fig. 2). Hence, our results indicate that differences in signal intensities do not appear to be due to up- or downregulation of specific HML-2 proviruses in the studied tissues. Other regulatory mechanisms may account for such differences.

Taken together, HML-2 expression analysis of brain samples indicates that quite a number of HML-2 proviruses are transcriptionally active in the human brain, independent of a benign or malignant condition. Based on cloning frequencies, which reflect to some extent relative expression levels, some HML-2 proviruses obviously contribute more to the HML-2 specific RNA pool than other proviruses, i.e., they are expressed at higher levels than others. Also, there is no obvious up- or downregulation of specific HML-2 proviruses when various conditions are regarded.

### HML-2 provirus expression in breast tissue

HML-2 transcripts were recently reported in normal and malignant mammary tissue (mamma-CA) [44,57,58]. We examined transcriptionally active HML-2 proviruses in mamma-CA samples that were available to us, as well as commercially available RNA from normal breast tissue. Based on 24 *gag *cDNAs pooled from 4 mamma-CA specimens, 21 *env *cDNAs from one mamma-CA, and 22 *gag *cDNAs and 19 *env *cDNAs from normal breast tissue, we identified 7 and 11, respectively, transcriptionally active HML-2 proviruses. In mamma-CA tissue about 71% (17/24) of *gag*-derived cDNAs were from provirus c1_B, while only 18% (2/22) of cDNAs were derived from that provirus in normal breast tissue. For *env*-derived cDNAs from c1_B, approx. 59% and 42% were from mamma-CA and normal breast tissue, respectively. While in mamma-CA no *gag *cDNA was derived from provirus c3_C, approx. 27% of *gag*-derived cDNAs and 10% of *env*-derived cDNAs were from that locus in normal breast tissue. cDNAs from other HML-2 proviruses were cloned at significantly lower frequencies [see Additional file 1]. Thus, based on cloning frequencies, besides basic expression of several HML-2 proviruses, provirus c1_B appears transcribed at somewhat higher levels in both mamma-CA and normal breast tissue, and provirus c3_C appears transcribed at somewhat higher levels in normal breast tissue. However, while our data provide an insight into HML-2 proviruses transcriptionally active in breast tissue analysis of a larger number of cDNAs is required to describe in greater detail the transcriptional pattern of HML-2 proviruses in normal and malignant breast tissue.

### HML-2 provirus expression in germ cell tumors

Because of the apparent association of HML-2 with GCT we aimed at better understanding expression patterns of HML-2 proviruses in GCT. We analyzed different seminoma samples, and atrophic, orchitic and normal testicular tissues as control samples. Seminoma tissue samples were chosen based on antibody titers against HML-2 Gag and Env proteins in respective tissue donors, measured by a previously established indirect immunofluorescence method [34]. Tissue donors either displayed antibodies against Gag and Env, only Env, or neither Gag nor Env.

**Figure 3:**
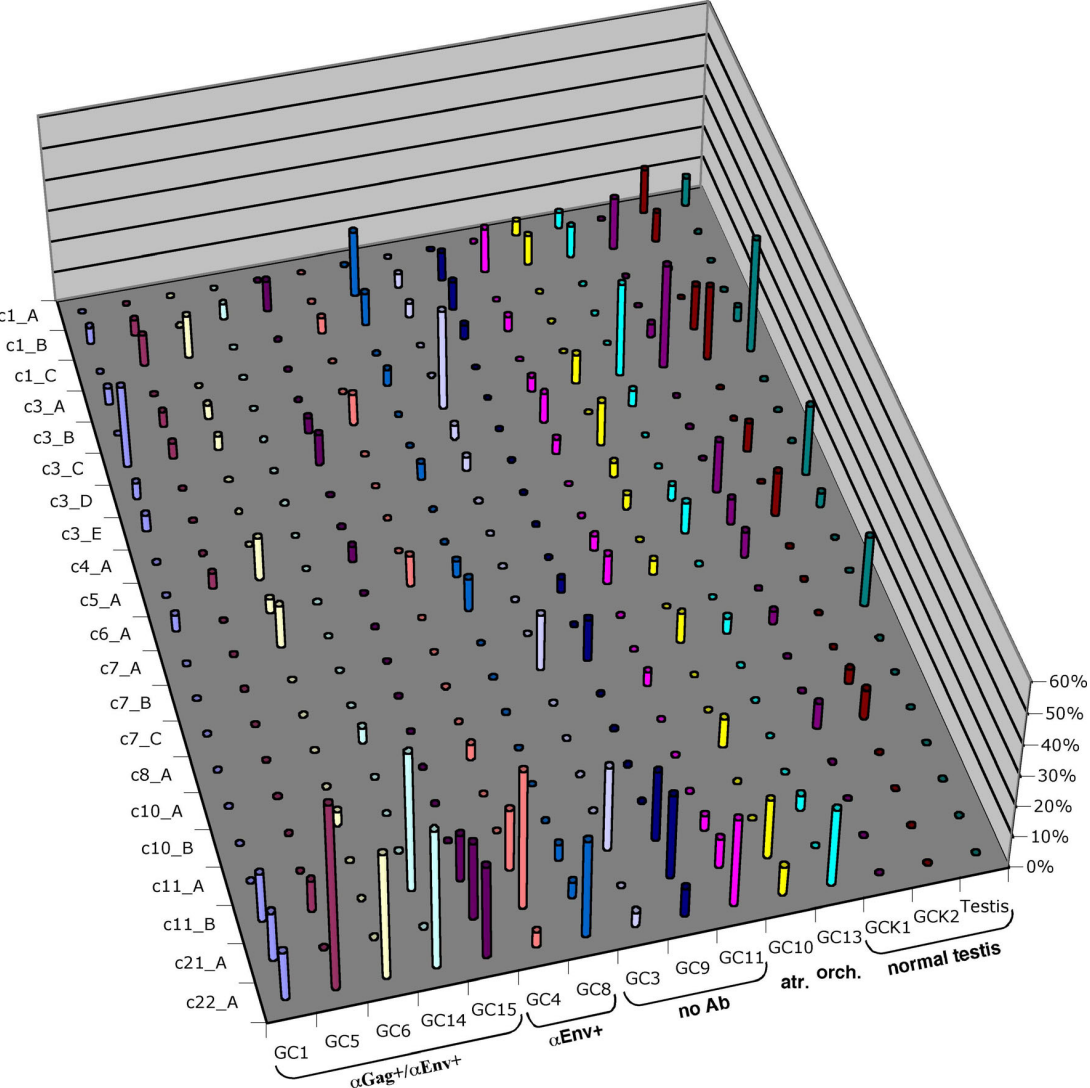
**Transcriptional activity of HERV-K(HML-2) proviruses in different germ cell tissues**. The various germ cell tissue specimens have been grouped based on presence of Gag and/or Env antibodies in patients (GC1 – GC8), specimens from atrophic and orchitic testis (GC10, GC13) and normal testis (GCK1 – Testis). Relative cloning frequencies for *gag*-derived cDNAs are given. See Table 1 for Gag, Env and Rec coding capacity of individual loci, and Fig. 2 and Additional file 1 for further details.

Overall, we identified 22 different HML-2 proviruses to be expressed in GCT tissues. Like for brain samples, cloning frequencies of cDNAs from individual proviruses, thus, presumably their expression levels, differed (Fig. 3). For provirus c3_C 15% of all *gag *and 21% of all *env *cDNAs generated from testicular tissues were derived from that provirus. Provirus c22_A was represented by approx. 20% of *gag *and 15% of *env *cDNAs. 11% of *gag *cDNAs but no *env *cDNAs were derived from provirus c11_B. About 19% of *env *cDNAs were derived from provirus c7_A whereas only 3.5% of *gag *cDNAs were derived from that locus. The same was found for provirus c1_B (17% *env *versus 8% *gag*).

While a number of proviruses displayed intermediate cloning frequencies cDNAs from some other proviruses were found rather rarely, potentially indicating low-level expression in the investigated testicular tissues. For instance, provirus c11_A was only represented by 1 testicular tissue-derived *env *cDNA, provirus c10_A by 2 *gag *cDNAs, and proviruses c12_A and c19_A by 3 *env *cDNAs each. Notably, the polymorphic status of provirus c8_A (also named HERV-K115) in the human population [29] may explain that only 2 *gag *cDNAs were found in two different tumor samples. Yet, provirus c8_A appears transcriptionally active, albeit low-level active when present.

On average, 9.9 (SD 1.7) proviruses were found expressed per seminoma sample, with 8.8 (SD 1,6), 10.7 (SD 1.2) and 11.5 (SD 0.7) proviruses found in samples from patients with antibodies against HML-2 Gag and Env, against Env and without antibodies, respectively. Samples from 3 normal testis displayed 8.3 (SD 1.5) transcribed proviruses. An atrophic and an orchitic testis sample displayed 14 and 9, respectively, active proviruses. Overall cDNA numbers for the various tissue samples were alike. As for the different tumor specimens, slight differences in the number of identified expressed proviruses did not appear to be due to expression of specific proviruses in one or the other tumor tissue (Fig. 3). Based on the present number of analyzed cDNAs, the different seminoma specimens seem to express the same set of HML-2 proviruses.

However, there is one notable difference between seminoma and normal testis. While provirus c22_A is found expressed in all seminoma samples, and in orchitic and atrophic testis samples, with an average relative cloning frequency of approx. 22% (range 4.5%–60% for *gag *cDNAs; 0%–70% for *env *cDNAs), no cDNA corresponding to provirus c22_A was found in the three normal testis samples, despite very similar numbers of analyzed cDNAs (Fig. 3). Provirus c22_A was also found in Tera-1 cells, but was rarely found in brain (3/493 cDNAs) and in normal mammary tissue (1/41 cDNAs). It thus appears that provirus c22_A is not expressed in normal testis or other investigated tissues, or it is expressed at very low level, but is activated or drastically upregulated in germ cell tumor cells (and also in other pathologic circumstances, namely atrophic and orchitic conditions). The same may be true for proviruses c11_B and c21_A but overall relative cloning frequencies of cDNAs from those proviruses were lower making it more likely that corresponding cDNAs were missed in normal testis.

**Figure 4:**
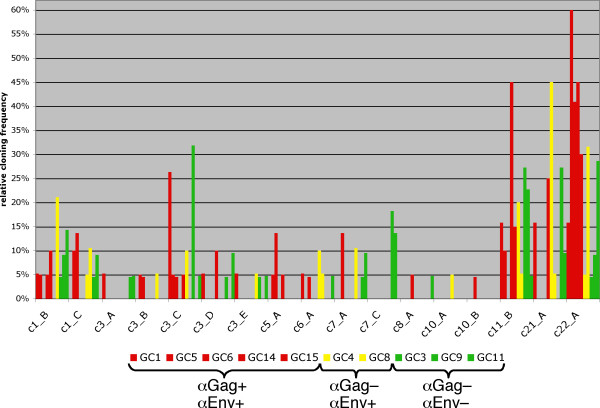
**No correlation between HML-2 provirus activity and HML-2 Gag and Env antibodies in GCT patients**. Relative cloning frequencies of *gag*-derived cDNAs from various HML-2 loci in malignant and pathologic testicular tissues are given. Only active HML-2 loci have been included. Results for tissue samples (GC1, 5, 6, 14, 15) from patients with antibodies against Gag and Env (αGag+/αEnv+) are shown as red bars. Yellow bars denote results for tissue samples (GC4, 8) from patients with Env but without Gag antibodies (αGag-/αEnv+). Green bars denote results for control tissue samples (GC3, 9, 11) from patients with neither Gag nor Env antibodies (αGag-/αEnv-). Note that none of the HML-2 loci appears exclusively expressed in all Gag and/or Env antibody-positive samples but not in antibody-negative control samples.

The presumably upregulated provirus c22_A harbors an ORF for Gag protein. Its prominent expression may explain strong Gag production in seminoma cells and in the Tera-1 cell line [34]. Still, other Gag-encoding HML-2 proviruses probably also contribute to the Gag production. Though, activation (or upregulation) of provirus c22_A or other Gag-encoding loci does not correlate with presence of antibodies in seminoma patients, as corresponding proviruses are found expressed in tumor samples from both Gag antibody positive and negative donors (Fig. 4). Likewise, there is no obvious correlation between expression of Env-encoding proviruses and presence of Env antibodies. Apparently, based on transcriptionally active HML-2 loci identified in our study, activation of single protein-encoding proviruses does not solely account for antibody production in GCT patients.

### Transcription of Rec expressing loci

Previously published results for the HML-2 encoded Rec protein point to an involvement of Rec in germ cell tumor development [35,36]. We therefore also analyzed the coding capacity of transcribed HML-2 proviruses for Rec. Recently, we identified HML-2 loci in the human genome with the potential to encode Rec, based on presence of required sequence elements and a Rec ORF. Rec mRNAs from some of the loci were identified in Tera-1 cells, in synovial cells and in the human EST database [42]. Seven transcriptionally active HML-2 proviruses (c3_D, c6_A, c7_A, c10_A, c10_B, c11_A, c12_A) were previously identified as candidates for Rec protein production (Table 1). Four of those proviruses (c6_A, c7_A, c11_A, c12_A) were also found expressed as *rec *mRNA in Tera-1 and synovial cells. In the present study, transcription of Rec encoding proviruses was found both in normal testis and in seminoma tissue (Fig. 3). Since most of the Rec encoding loci are among the low-level expressed HML-2 loci, corresponding cDNAs were detected at low frequencies and not in all tissue samples. cDNAs from 5 Rec-encoding proviruses (c3_D, c6_A, c7_A, c10_A, c11_A) were also found in 5 different brain samples, likewise at low frequencies and not in all tissue samples. There was no obvious expression of one or several Rec encoding proviruses exclusively in testicular tumor tissues (Table 1, Additional file 1). While Rec-encoding HML-2 proviruses are expressed in testicular tissue, our present data do not allow concluding on the role of Rec expression in germ cell tumorigenesis.

### Analysis of HML-2 specific human ESTs

**Figure 5:**
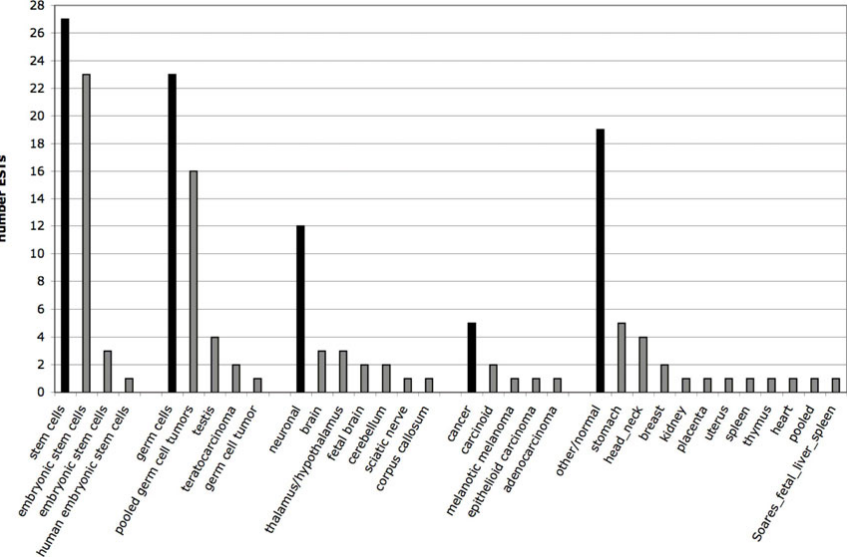
**Summary of tissue origins of HERV-K(HML-2)-derived ESTs in the human section of dbEST**. Information on tissue origins was compiled from EST sequence entries. Source tissues were combined in appropriate groups. Black bars indicate the total number of ESTs per source tissue group. Grey bars further indicate the number of ESTs from individual source tissues within a source tissue group. Tissue designations are as given in the EST sequence entries. Note that some ESTs lacked information on source tissues.

We compared our results with data provided in the human section of dbEST. We performed a BLAST-search in the human dbEST using a 7537 bp HERV-K(HML-2.HOM) *gag*-*pro*-*pol*-*env *region (that basically excluded the LTRs) as probe sequence, and employing standard parameters. The search yielded significant hits to, in total, 109 different EST entries. We retrieved those EST entries and compiled information on source tissues (Fig. 5). In total, 27 ESTs were derived from stem cells, 23 were derived from germ cells, 12 were derived from neuronal tissues, 5 from cancerous tissues, and 19 were derived from a number of other, normal human tissues. Other ESTs lacked information on the source tissue. As for ESTs from germ cell tissue, 16 were derived from "pooled germ cell tumors", and 1, 2 and 4 ESTs were derived from "germ cell tumor", "teratocarcinoma" and "testis", respectively. There were no ESTs from brain tumors among the retrieved ESTs. For reasons discussed below, we did not further analyze the proviral origin of the EST sequences.

## Discussion

Every human tissue harbors HERV transcripts. While overall transcriptional activities of various HERV families have been studied in more detail, little is known about transcriptional activities of specific HERV loci in benign and malignant tissues. For instance, are only a few or a greater number of loci for a given HERV family and tissue transcriptionally active? Is the expression of specific HERV loci up- and downregulated depending on the cellular condition? Given the significant amount of HERV sequences in the human genome a better understanding of the regulation of HERV transcription appears crucial for better understanding transcriptional regulation in the human genome as a whole. Besides, alterations in transcriptional activities of HERV loci may provide important insight into epigenetic alterations in corresponding genomic regions, potentially affecting critical cellular genes located in those regions. In the following, we will also emphasize the need for larger scale and specialized studies to adequately address those issues.

Identification of transcriptionally active HERVs is a prerequisite for better understanding regulation of HERV transcription. We therefore aimed in the present study to identify transcriptionally active proviruses of the HERV-K(HML-2) family that is of importance from an evolutionary and a clinical perspective. The strategy for identifying transcriptionally active HML-2 sequences is relatively straightforward. Because the various HML-2 loci in the human genome accumulated random mutations over time each HML-2 locus harbors private nucleotide differences (Fig. 1). Thus, cDNAs generated from RNA transcripts from a particular HML-2 provirus should be identical in sequence to the original provirus. In practice, this may not hold true because of RT-PCR errors. Also, SNPs within HML-2 sequences may introduce differences when compared to the human reference sequence. Previous work already indicated SNPs within HML-2 coding regions [28,59]. However, the best cDNA-provirus sequence match is still expected to be the correct one if matches to other proviruses display clearly more differences. Assignment of HML-2 cDNA is further complicated by *ex vivo *recombinations between transcripts from different proviruses that inevitably occur during cDNA generation [53]. In the present study, we chose to exclude cDNA sequences that displayed 18 or more nt differences to the best match. While the great majority of cDNAs displayed between zero and 5 nt differences [53] it is likely that there was a limited number of recombinant cDNAs among the cDNAs with less than 18 nt differences. Actually, a SAGE (serial analysis of gene expression)-like strategy could be applied to such recombinant cDNAs if it was possible to unambiguously assign each recombined portion to a particular provirus. Since HML-2 sequences are relatively similar in sequence, proper assignment seems difficult or impossible, but a SAGE-like strategy may be feasible for other, evolutionarily older and thus more diverged HERV families.

Since little is known about transcription of individual HERV sequences in various human tissues, a greater number of tissues would be of interest for initial studies. We investigated in the present study in more detail normal and pathologic germ cell tissue because of the association of HML-2 with GCT [1,32], and brain tissues because of previous results indicating transcriptional differences between tissue samples from patients with neuropsychiatric disorders [56].

Our study identified, in total, 23 different HML-2 proviruses as transcriptionally active in the studied tissues. Several HML-2 proviruses appeared transcribed in every investigated tissue. We stress in this context that our method is not intended to provide quantitative information as to overall transcriptional levels of HML-2 sequences in different tissues but to identify those loci that are transcriptionally active at all. In any case, among the transcriptionally active proviruses were structurally intact, full-length proviruses and proviruses lacking internal or lateral proviral regions. Both type 1 and type 2 proviruses, differing by a 292 bp sequence within the *pol*-*env *boundary, were found transcribed. Notably, few loci (c7_C, c10_B, c19_A) lack the 5'LTR as the classical proviral promoter. Hitherto unknown flanking promoters obviously result in transcription of those HML-2 sequences. Given results from the ENCODE project, specifically the density of initiators of transcription in the human genome [51,52], it seems plausible that there are promoters located nearby. Alternatively, as locus c10_B is located within a gene intron, rare splicing events might have produced c10_B-harboring transcripts. For locus c7_C, located closely downstream from the SSBP1 gene, read-through events might have produced c7_C-harboring transcripts.

It is likely that there are more than 23 transcriptionally active HML-2 proviruses. Oligonucleotides employed for PCR following the RT step display varying amounts of mismatches to HML-2 loci in the human genome. Transcriptionally active HML-2 loci tended to have less mismatches, however, several seemingly inactive loci displayed as few mismatches as active loci. One or both primer regions are missing in some of the HML-2 loci [see Additional file 3]. Transcripts from some proviruses were indeed detected only as *gag*- or *env*-derived cDNAs. While transcripts from several HML-2 loci with one or two mismatches to the primers were eventually cloned as cDNA, it is difficult to decide for other loci whether they were not expressed or could not be efficiently cloned as cDNA for technical reasons. In fact, a type 1 provirus on human chromosome 3, previously termed HERV-K(II), was not identified in our analysis, despite the fact that it is known to produce np9 mRNA [[45,60], Kehr et al. unpublished]. Transcripts from that provirus were probably not detected because of 25% and 20% sequence differences to *gag *and *env*, respectively, reverse primers. In the course of larger scale studies degenerate oligonucleotides or sets of oligonucleotides representing proviral sequence variants should be employed.

Another point concerns relative cloning frequencies. While HML-2 sequences expressed at higher levels are more likely to be cloned as cDNA, many other HML-2 loci may be transcribed at much lower levels and thus their cDNA would only appear when many more cDNA sequences per tissue sample were analyzed. We observed such differences between tissues for several HML-2 loci. Considering current sequencing technologies, it should be quite feasible to generate significantly more cDNA sequences per tissue sample in the course of a larger study.

Different splicing efficiencies for HML-2 transcripts may also affect cloning likelihoods of corresponding proviral transcripts if, for instance, full-length transcripts are efficiently spliced down to *env *or *rec *mRNA, making it less likely to catch *gag *portions as cDNA, as opposed to splicing-deficient transcripts. However, besides the fact that type 1 proviruses lack splice signals present in type 2 proviruses, yet, type 1 transcripts still being spliced [61,62], there is currently no information about splicing efficiencies of transcripts from different HML-2 loci.

Furthermore, the polymorphic nature of several potentially transcriptionally active HML-2 proviruses will result in lack or potentially reduced amounts of corresponding transcripts for tissue samples from some human donors. In fact, six of the proviruses identified in our study (c1_A, c6_A, c7_A, c8_A, c11_A, c12_A) have been described polymorphic in the human population. Two of those loci (c1_A and c8_A) represent presence/absence alleles, the other alleles are full-length (either tandem or single) proviruses, solitary LTRs and/or (empty) preintegration sites [26].

Based on cloning frequencies, a limited number of HML-2 proviruses appear transcribed at higher levels in the investigated brain and testicular tissues than other proviruses. It is currently not clear whether the former proviruses are also transcribed at higher levels in other human tissues, potentially even showing ubiquitous expression. Investigation of other human tissues in the course of a larger project should clarify that point.

GCTs, especially seminomas, display significantly upregulated HML-2 expression, both on the RNA and protein level. Variable HML-2 expression was previously also observed in different human brain samples [56]. On the assumption that relevant proviruses were not missed in our analysis, our results indicate that observed differences in expression levels were not primarily due to drastic upregulation of single HML-2 proviruses but rather due to global upregulation of HML-2 transcription. This could be explained by differential expression of factors involved in the regulation of HML-2 sequences, such as transcription factors. Deregulated levels of such factors in GCT cells could result in the activation of HML-2 sequences. As for previously observed interindividual differences in HML-2 expression levels [56], such differences have also been observed in the human population for cellular genes, and sets of genes appear regulated by hitherto unidentified elements (gene products) in defined genome regions [63-66]. If regulators of HML-2 transcription were differentially expressed in humans expression levels of those regulators could indirectly result in differential HML-2 expression levels.

The Gag-encoding provirus c22_A appears strongly upregulated in pathologic versus normal testicular tissue, and may thus contribute to high Gag protein levels in GCT. However, the results from our study also indicate that presence of HML-2 Gag and Env antibodies in GCT patients is not correlated with expression of specific HML-2 proviruses, as several Gag- and Env-encoding HML-2 proviruses, among them c22_A, are transcriptionally active in both antibody positive and negative GCT patients. Generation of autoantibodies is currently little understood. It has been associated with increased expression of antigens. Immunogenic antigens, i.e. antigens that cause a humoral immune response, can also stem from genes altered by specific mutations including chromosome alterations and epigenetic DNA modifications. Likewise, spliced gene products can give rise to an immune response. Posttranslational modifications, such as altered protein folding and processing, may also result in immunogenic antigens. Finally, normal non-altered antigens can elicit an autoantibody response when expressed in specific tissues [for a review, see [67]].

Two research groups [23,24] recently described engineered HML-2 proviral sequences that are replication-competent and infectious. An additional engineered proviral sequence, consisting of portions of so-called HERV-K109, HERV-K108 and HERV-K115 proviruses, was reported to generate an infectious retrovirus as well [24]. The authors argued that recombination between those proviruses *in vivo *could generate a functional HERV-K(HML-2) variant. We note that those three proviruses, named c6_A, c7_A and c8_A in our study, have been identified by us as transcriptionally active in germ cell tissue. For all three proviruses have been described polymorphic in the human population, only some human individuals will harbor all three proviruses, though. In any case, for individuals harboring all three, transcripts from those proviruses would be present and could serve as substrates for recombination events when transcripts were reverse transcribed.

Large-scale cDNA sequencing projects have so far generated about 8.1 million human expressed sequence tag (EST) entries . HML-2 provirus-derived ESTs are also found in dbEST. However, our initial analysis of HML-2 specific ESTs clearly shows that there is insufficient information in dbEST to comprehensively describe HML-2 proviral expression patterns. Our initial probing of dbEST with a representative HML-2 *gag*-*pro*-*pol*-*env *sequence yielded only 109 significant hits. Breaking down those hits to specific tissues resulted in a maximum of 27 ESTs that were derived from stem cells, only 23 ESTs were derived from germ cells. It is likely that several of those 23 ESTs stem from the same HML-2 proviruses. Considering the usually poor sequence quality of ESTs, proviral assignment of ESTs may often be ambiguous. Moreover, HML-2 ESTs stem from very few source tissues. Indeed, few HML-2 ESTs in dbEST stem from tissues in which HML-2 is clinically relevant, i.e. germ cell tumors. Such lack of HML-2 ESTs for other tissues erroneously indicates that HML-2 is not transcribed in those tissues. Though, more detailed analysis of EST data does not provide significantly more and better information. Stauffer et al. [48] recently reported such a more detailed analysis of HERV-derived ESTs, among them HML-2-specific ESTs. They identified 143 non-normalized, non-subtracted ESTs matching the HML-2 family. About a third of the ESTs could not be assigned unambiguously to specific HML-2 proviruses. In total, 8 HML-2 proviruses (and 3 ambiguous ones) were identified as transcriptionally active. Of those, 2 (or 4 when including the ambiguous ones) were also identified in our study (Table 1). In other words, our study detected at least 19 transcriptionally active HML-2 proviruses that were not identified in the detailed EST study by Stauffer et al. A more recent analysis by Oja et al. [49] reported about 300 HML-2-specific ESTs. However, it seems clear that 300 HML-2 ESTs are still insufficient to describe HML-2 expression patterns in, for instance, human benign and malignant tissues, and depending on cellular conditions and stimuli. It is well conceivable that conclusions for HML-2-specific ESTs are true for ESTs from other HERV families as well. One can also predict that (overall much smaller) non-human EST datasets are likewise insufficient for studying transcriptional activity of ERVs in other species. Thus, EST data are insufficient to describe (H)ERV transcription.

Buzdin et al. [43] recently reported results from an experimental strategy, named GREM (Genome Repeat Expression Monitor) that is able to specifically amplify HERV transcripts. A total of 22 HML-2 proviruses were reported transcribed in parenchyma and/or seminoma. Of those proviruses, 16 were also identified in our study (Table 1), 7 and 6 proviruses were found exclusively in our or in the study of Buzdin et al., respectively. Thus, specialized generation and analysis of HERV-specific transcripts in the study of Buzdin et al. likewise identified many more transcriptionally active HML-2 proviruses than the very limited number of ESTs in dbEST.

We conclude that a specialized HERV transcript sequencing project, a *HERV Transcriptome Project*, will be required to comprehend transcriptional activity of HERVs. Bearing in mind the proportion of HERV sequences, and recent findings regarding the extent of transcription in the human genome, a HERV Transcriptome Project could provide crucial information for better understanding transcriptional regulation not only of HERVs but also of the human genome in general. For instance, many of the HERV sequences could function as transcription initiators and could thus contribute to the pervasive transcription of the human genome [51]. Considering previously reported differences in HERV expression patterns between human tissues, transcriptional activities of individual HERV proviruses are very likely significantly different between tissues as well. Moreover, a HERV Transcriptome Project could significantly contribute to a better understanding of proposed roles of HERV sequences in various human diseases if relevant source tissues were analyzed in greater detail. From that point of view, it will be important to better understand cellular conditions under which HERV sequences are activated or repressed [for instance, see ref. [68]].

The current study provides an initial insight into the transcriptional activity of a clinically relevant HERV family in selected human tissues. We believe that strategies to specifically generate significantly greater numbers of HERV-specific cDNAs from human tissues and selected cellular conditions can be established in a straightforward fashion. Current sequencing technologies can produce significant amounts of HERV cDNA sequences in a short time that can then be used to identify transcriptionally active HERV loci. We furthermore believe that a HERV Transcriptome Project would best be pursued in a collaborative fashion, that is, the project should be open to researchers proposing analysis of specific HERV families for tissues or cellular conditions of specific interest.

## Conclusion

By assigning ~1500 HML-2 cDNA sequences to individual genomic loci we showed that a considerable number of HERV-K(HML-2) loci is transcribed in the investigated human tissues. Some loci appear transcribed at higher levels than others and some loci are active in several tissue types. As for the involvement of HML-2 in GCT, our results point to a provirus on human chromosome 22q11.21 being activated in pathologic germ cell tissue that may significantly contribute to observed high Gag protein levels in GCT cells. However, antibodies against HML-2 Gag and Env in GCT patients do not appear to result from activation of specific HML-2 loci. Other mechanisms seem to trigger HML-2-specific immune responses. As HML-2 Rec-encoding loci are active in non-GCT tissues, sole expression of Rec in GCT cells is not the main reason for its potential involvement in GCT development. EST data generated to date are clearly insufficient to describe in greater detail transcriptional activities of HML-2 and other HERVs in human tissues and cell types. Our analysis thus also illustrates that larger-scale analysis will be essential to comprehensively describe transcriptional activities of individual HERV loci. Considering the amount of HERV sequences in the human genome, and ERV sequences in other genomes, studying the contribution of (H)ERVs to species' transcriptomes is vital to better comprehend transcription in genomes in general. A specialized (H)ERV Transcriptome Project is needed.

## Materials and methods

### Tissue samples

Tissue samples from tumors (brain, breast, lung, testis) and orchitic and atrophic testis were obtained from surgical procedures. Tumor tissue specimens from testicular cancer were obtained from previously untreated patients. Following orchiectomy, tissue samples were dissected from macroscopically supposed tumor lesions, snap frozen, and stored at -80°C. Only samples containing >80% tumor cells were included in the study. Tissues were obtained with informed consent of patients according to the Declaration of Helsinki. Postmortem brain tissue samples were obtained from the Stanley Foundation Brain Collection (Bethesda, MD, USA) [69]. RNA from normal testis and breast tissue was obtained from Stratagene Inc. Germ cell tumor-derived cell line Tera-1 and mammary carcinoma cell line T47D were cultured in DMEM and RPMI-1640, respectively, media supplemented with 10% fetal calf serum in a humidified 5% CO_2 _atmosphere.

### cDNA generation and sequencing

RNA was isolated using Trizol (Invitrogen), following the manufacturer's recommendation. RNA was subsequently treated with DNase I (Roche) to remove residual traces of genomic DNA. Complete removal of DNA was verified by an Alu element-specific PCR [70]. DNA-free RNA was used for subsequent cDNA generation, employing the Omniscript RT Kit (Qiagen) and random hexanucleotide primers following the manufacturer's recommendations. For each cDNA preparation control reactions without reverse transcriptase were included. Subsequent PCR employed the following HERV-K(HML-2) specific primers to generate *gag *and *env *gene-derived PCR products: gag_plus (5'GGCCATCAGAGTCTAAACCACG3'; nt 1626–1647 in HERV-K(HML-2.HOM); Genbank accession number AF074086); gag_minus (5'GCAGCCCTATTTCTTCGGACC3'; nt 2242–2262); env8146F (5'AATGAGTCTGAGCATCACTGGG3'; nt 8146–8167), and env8665R (5'CCATTCAACTCTGAGTGGACACAG3'; nt 8665–8688). The PCR mix consisted of 0.5 μM of each PCR primer; 1.5 mM MgCl_2_; 0.2 mM dNTPs; 2.5 units Taq polymerase (Invitrogen); 1 × PCR buffer in a total volume of 50 μl. PCR cycling conditions were as follows: initial denaturation 5 min. 94°C; 40 cycles 1 min. 94°C; 45 sec. 57°C; 1 min. 72°C; final elongation 10 min. 72°C. Generation of cDNA from brain tissues from normal, bipolar and schizophrenic conditions was described previously [56]. RT-PCR products were subsequently purified using spin prep columns (PeqLab), ligated into the pGEM T-Easy vector (Promega) and transformed into DH5α or TOP10F' bacterial cells. Insert-containing clones were identified by standard colony-PCR using above primer combinations. Plasmid DNA of positive clones was isolated using a standard column procedure (QIAprep Spin Miniprep Kit; Qiagen). Sequences of cloned cDNAs were obtained using vector-specific sequencing primers and an Applied Biosystems 3730 × Capillary Sequencer (Institut für Immunologie und Genetik, Kaiserslautern, Germany). Quality of sequences was assessed using CodonCode Aligner (CodonCode Corporation, Dedham, MA, USA) and FinchTV (Geospiza Inc., Seattle, WA, USA) and corrected if necessary. Poor quality sequence reads were excluded.

### cDNA sequence assignment to proviral loci

Sequences of HML-2 proviral loci, including remnants of proviral loci, were collected from the human genome sequence as given at the Human Genome Browser March 2006 version [55] by using the HERV-K(HML-2.HOM) sequence [54] as probe for BLAT searches [71]. Matching sequence portions plus flanking sequences were retrieved, aligned using DiAlign [72] and MAFFT [73] and the alignment was manually optimized using Se-Al . *Gag *and *env *subregions were extracted from the alignment and used as reference sequences for further analysis. Locus-Assigner is an in-house Bio-Python script for assigning experimental HERV cDNA sequences to HERV reference sequences. The script is available from the authors on request. The strategy of assigning cDNA sequences to specific proviruses was based on private nucleotide differences between the various HML-2 sequences (Fig. 1). Private means one or several nucleotides that are characteristic for one HML-2 locus when compared to all other loci. Ideally, a cDNA will be identical to the corresponding proviral sequence that generated the original transcript, but dissimilar to all other proviral loci. Locus-Assigner uses as input an experimental cDNA sequence file and a reference sequence file and generates all possible pairs of experimental cDNA sequences and reference sequences that are saved as individual fasta files. Locus-Assigner then generates pairwise sequence alignments for each cDNA sequence/reference sequence pair using CLUSTAL W. Pairwise alignments are saved as separate files. For each of those files, the total amount of nt differences between the aligned pair of sequences is calculated. Mismatches due to start and end gaps were ignored, as some cDNA and reference sequences lacked portions at the 5' or 3' end. For each experimental sequence Locus-Assigner then creates a tab-separated file summarizing detected nucleotide differences compared to all reference sequences. Using appropriate software, nt differences can be sorted, thus revealing for each cDNA sequence the most closely related reference locus, that is, the provirus that most likely generated the original transcript. The Locus-Assigner script was verified with a number of test sequences that were run against the HML-2 reference sequence dataset. Test sequences either derived from genomic HML-2 sequences or from cDNA sequences with known amounts of nt differences, 5', 3', and/or internal gaps. Locus-Assigner results were further verified by comparison with results from probing test sequences by BLAT at the UCSC Human Genome Browser, by visual inspection of alignments and by checking several of the Locus-Assigner cDNA/HML-2 locus assignments. cDNA sequences with 18 or more mismatches with their best matching reference sequence were excluded from further analysis because they very likely represent recombined cDNAs from different proviral transcripts that arose *ex vivo *during cDNA generation [53].

## Authors' contributions

AF, OF, AR, MS, and EMa contributed to molecular genetic and sequence analysis. AF compiled data and wrote software. BK and BW prepared germ cell tissue samples. NM–L, WS, CL–M and EMe conceived and financed the study. JM conceived and financed the study, compiled and analyzed data, and wrote the paper. All authors read and approved the final manuscript.

## Competing interests

The authors declare that they have no competing interests.

## Supplementary Material

Additional File 1 Cloning frequencies. Summary of relative and absolute cloning frequencies of cDNAs from different HERV-K(HML-2) loci. Click here for file

Additional file 2 HML-2 locus structures. Dot plot analysis of transcriptionally active HERV-K(HML-2) loci depicting structures of individual loci. Click here for file

Additional file 3 Primer-template comparisons. Sequence comparison depicting (mis)matches between utilized *gag *and *env *PCR primers and primer binding regions in HML-2 loci. Click here for file
