# Supplementary material for: Expression patterns of transcribed human endogenous retrovirus HERV-K(HML-2) loci in human tissues and the need for a HERV Transcriptome Project
Source: BMC Genomics. 2008 Jul 29;9:354. doi: 10.1186/1471-2164-9-354 (PMC2525661; doi:10.1186/1471-2164-9-354)
Supplement: Additional file 2 — HML-2 locus structures. Dot plot analysis of transcriptionally active HERV-K(HML-2) loci depicting structures of individual loci. [file 1471-2164-9-354-S2.pdf]

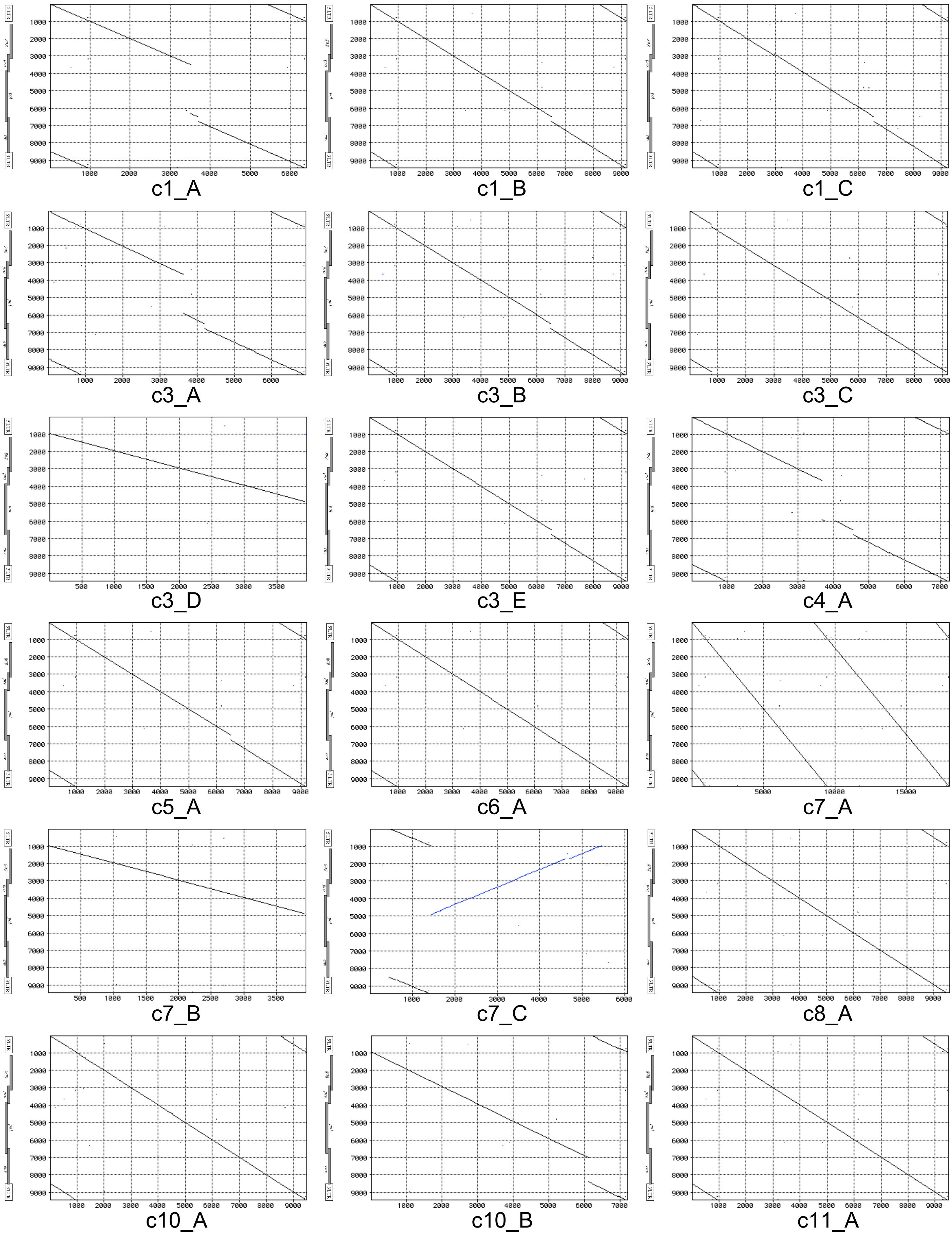
c1_A
c1_B
c1_C
c3_A
c3_B
c3_C
c3_D
c3_E
c4_A
c5_A
c6_A
c7_A
c7_B
c7_C
c8_A
c10_A
c10_B
c11_A

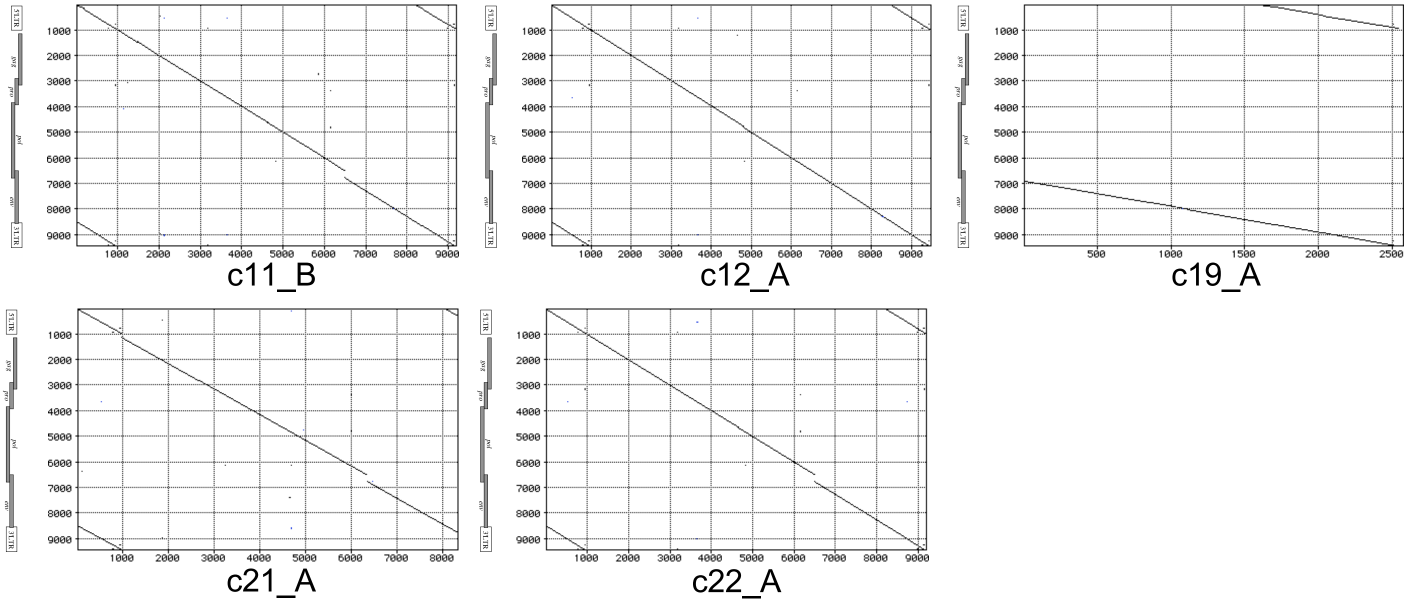

<u>Flockerzi et al. Additional Figure 1:</u> Pustell matrix comparisons of HERV-K(HML-2) proviruses identified as transcriptionally active in this study. Proviral sequences were compared to the previously published HERV-K(HML-2.HOM) sequence. Location of LTR and proviral *gag*, *pro*, *pol* and *env* genes within the latter sequence is indicated. The following parameters were applied for comparisons: Window size = 30 nt; Min. % Score = 55; Hash Value = 6; Jump = 1; Strands = both. Note that provirus c7_A—the actual HERV-K(HML-2.HOM) provirus—is represented in tandem structure in the human genome reference sequence. HERV-K(HML-2) type 1 loci display a gap at nt 6500 with respect to the HERV-K(HML-2.HOM) sequence, as opposed to type 2 loci. Provirus sequences were reverse complemented prior to comparison if located in antisense on the respective human chromosome. The following parameters were applied for comparisons: Window size = 30 nt; Min. % Score = 55; Hash Value = 6; Jump = 1; Strands = both. Thus, each position in one sequence that shares on the DNA level at least 55% similarity in a 30 nt window to the other sequence is marked by a dot, and producing a line for longer stretches of DNA.
