# Supplementary material for: Expression patterns of transcribed human endogenous retrovirus HERV-K(HML-2) loci in human tissues and the need for a HERV Transcriptome Project
Source: BMC Genomics. 2008 Jul 29;9:354. doi: 10.1186/1471-2164-9-354 (PMC2525661; doi:10.1186/1471-2164-9-354)
Supplement: Additional file 3 — Primer-template comparisons. Sequence comparison depicting (mis)matches between utilized gag and env PCR primers and primer binding regions in HML-2 loci. [file 1471-2164-9-354-S3.pdf]

A

	gag_plus	*gag* cDNA	gag_minus (rev. compl.)
	GGCCATC-AGAGTCTAAACCACG		GGTCCGAAGAAATAGGGCTGC
**c1_A**	.......-...............	yes	....
**c1_B**	.......-.............T.	yes	....
**c1_C**	......T-...............	yes	....
**c3_A**	.......-...............	yes	........A............
**c3_B**	.......-...............	yes	....
**c3_C**	.......-.............T.	yes	....
**c3_D**	.......-...............	yes	....T................
**c3_E**	.......-...............	yes	....
**c4_A**	.......-......C........	yes	....
**c5_A**	.......-...............	yes	....
**c6_A**	.......-...............	yes	....
**c7_A**	.......-...............	yes	....
**c7_B**	.......-...............	yes	....
**c7_C**	.......-...............	yes	....T.......C........
**c8_A**	.......-...............	yes	....
**c10_A**	.......-.A.............	yes	....T................
**c10_B**	.......-.........G.....	yes	....
**c11_A**	.......-.............T.	yes	....
**c11_B**	.......-...............	yes	....
**c12_A**	.......-...............		.....A...............
**c19_A**	-----------------------		---------------------
**c21_A**	.....C.-...............	yes	..............A......
**c22_A**	.......-...............	yes	....
1p36.21	...T.C.-....C.........C		..CAT...A....CA....AT
1p36.21	...T.C.-....C.........A		..CA....A....CA....AT
3p12.3	.......-.....T..G....T.		...AT.G.A....CA....AT
3q12.3	.......-...............		...A....A....CA....A.
4p16.2	.......-.....T..G......		...AT...A....CA....A.
4p16.3	-----------------------		...A....A....CA....A.
4p16.1	.......-.....T..G....A.		...AT...A....CA....A.
4q32.2	.......-...............		....
5p11	......A-....CA.......T.		....
5p13.3	......T-.............T.		....
5q33.2	..T....-.....T.........		...AA.G.A....CA....A.
6p21.1	.......-....C..........		...A....A....CA..T.A.
6p11.2	......T-G.G.CTA.......A		---------------------
6p22.1	....G..-..............A		.A...A..A............
8p22	-----------------------		A.AT.A..A...CC.T.....
8p23.1	.A.....-.....T..G.A....		...A....A....CA....A.
8p23.1	.A.....-.....T..G......		...A....A....CA....A.
8p23.1	.......-.....T..G......		...A....A....CA....A.
8q11.1	....T.T-G.G.CTA........		---------------------
8q24.3	.......-...............		.....----------------
8q24.3	.......-.....T..G......		...A..G.A....CA....A.
9q34.11	......T-G.G.CTAC......A		---------------------
9q34.3	.......-...............		........A............
11p15.4	.......-.....T..G......		.C.A....A....CA....A.
11q12.1	......TTG.G.CTA......G.		---------------------
11q12.3	.......-.............T.		....T....T...........
12q24.33	...TG.T-G.G..TA...G....		---------------------
14q11.2	.T.....-.....T..G......		...AT.G.A....CA....A.
16p13.3	.......-..............A		---------------------
17p13.1	-----------------------		A.AT....A...CC.T.....
19p12	.......-...............		....
19p12	.......-.............T.		.....A..A............
19p12	.......-...............		T...T................
19q12	.......-...............		....
19q13.12	.......-...............		.....A..A............
19q13.41	..T...A-G.G.CTA........		---------------------
20q11.22	.......-.........C.....		.A....C.A.........C..
22q11.23	.......-....C..........		...A....A....CA....A.
Xq28	....G..-..G.CTA........		---------------------
Yp11.2	.......-.....T..G..G.T.		T..A.AG.A....CA....A.

B

	env8146f	*env* cDNA	env8665r (rev. compl.)
	AATGAGTCTGAGCATCACTGGG		CTGTGTC-CACTCAGAGTTGAATGG
**c1_A**	....	yes	.......-A..........A.....
**c1_B**	....	yes	.......-A......G.........
**c1_C**	....		.......AA......G...A.....
**c3_A**	....	yes	.......-.......G...A.....
**c3_B**	....	yes	.......-A..........A.....
**c3_C**	...................A..	yes	.......-A................
**c3_D**	----------------------		-------------------------
**c3_E**	....		..C....-A..........A.....
**c4_A**	....		.......-A......G...A.....
**c5_A**	....	yes	.......-A......G.........
**c6_A**	....	yes	.......-A..........A.....
**c7_A**	....	yes	.......-.................
**c7_B**	GT..G.CGCC..ATGT.GG..-		-------------------------
**c7_C**	GG.TG.G.GCCAG..G.AG..-		-------------------------
**c8_A**	.....C................		.......-A......G.........
**c10_A**	....	yes	.......-A......G...A.....
**c10_B**	----------------------		-------------------------
**c11_A**	....	yes	.......-A................
**c11_B**	....		.......AA......G...A.....
**c12_A**	....	yes	.......-A..........A.....
**c19_A**	...................A..	yes	.......-.......G.........
**c21_A**	.........A.........A..	yes	.......-A................
**c22_A**	....	yes	.......-A..........A.....
1p36.21	.................A....		T...A.G-G.A..GAG...T..G..
1p34.3	....		.......-G.....AG...T.....
1q21.2	----------------------		.......-A......G...A.....
1q24.1	....		T.-.A.G-G.A...AG...T..G..
1q32.2	....		.......-A......G...A.....
1q43	....		....A.G-G.A...AG...T..G..
2q21.1	....		.......AA......G...A.....
2q32.1	----------------------		.......------.AGA..T.....
3p12.3	----------------------		T...A.G-A.A...AG...T..G..
3p12.3	TGATG..TCC....ATGGAAAA		-------------------------
3p12.3	..C..A.....A..........		T...A.A-A.A...AG...T..G..
3q12.3	....		.......AA......G...A.....
3q21.2	----------------------		T...A.G-A.A...AG...T..G..
4p16.2	.....A.....A..........		-------------------------
4p16.2	----------------------		T...A.G-A.A...AG...T..GAC
4p16.1	----------------------		T...A.G-A.A...AG...T..G..
4p16.1	----------------------		T...A.G-A.A...AG...T..G..
4p16.1	.....A.....A..........		T...A.A-A.A...AG...T..G..
5p11	........A...T....G..T.		.......-A......G...AC....
5p13.3	....		.......-A........A.......
5q33.2	...A.A.....A..........		T..CA.G-A.A...AG...T..G..
6p22.1	....		......T-G.....AG...T.G...
6p21.1	....		T...A.G-G.A...AG...T..G..
6p11.2	----------------------		T...A.A-A.A...AG...T..G..
6q25.1	.............C........		....A..-A.A...AG...T.....
7q11.21	.....C.....A.....A....		T...A.G-G.A...AG...T..G..
8p22	....TTA....A.....G....		-------------------------
8p23.1	----------------------		T...A.G-A.A...AG...T..G.C
8p23.1	----------------------		T...A.G-A.A...AG...T..G.C
8p23.1	.....A.....A..G.......		T...A.A-A.A...AG...TG.G..
8p23.1	----------------------		T...A.G-A.A...AG...T..G.C
8p23.1	----------------------		T...A.G-A.A...AG...T..G.C
8p23.1	.....A.....A..G.......		T...A.A-A.A...AG...TG.G..
8p23.1	.....A.....A..G.......		T...A.A-A.A...AG...TG.G..
8q11.1	.....A.....A..........		T...A.G-G.A...AG.C.T..G..
8q24.3	....		.......-A..........A.....
8q24.3	.....A.....A..........		T...A.G-A.G...AG...T..G..
9q34.11	.....A.....A..........		T...C.G-G.A...AG...T..G..
9q34.3	----------------------		......T-A.....AG...TG.G..
11p15.4	.....AC....A..........		T...C.A-A.A...AG...T..G..
11q12.1	....................A.		T...A.G-G.A...AG...T..G..
11q12.3	----------------------		TC..A.G-A.A...AG...T..G..
11q13.2	----------------------		TC..A.G-A.A...AG...T..G..
11q13.4	----------------------		T...A.G-A.A...AG...T..G..
12q24.33	.....A.....A..........		T...A.A-A.A...AT...T..G..
14q11.2	----------------------		T...A.G-A.A...AG...T..G..
16p11.2	....		.......-A..........A.....
16p13.3	TCCCC.G...GCTCAGT.A.T-		-------------------------
19p12	....		.......-A................
19p12	....		......T-G.T...AG...T.....
19p12	....		.......-A......G...A.....
19p12	....		......T-G.....AG...T.....
19q12	....		.......-A..........A.....
19q13.12	....		-------------------------
19q13.41	.................T....		....A.T-G.A...AG...T.....
20q11.22	....		....A.T-G.....AG...T..-..
22q11.23	....		....---------.AG...T..G..
Yp11.2	.....A...A.A..........		T...A.G-G.A...AG...A..G..

Flockerzi et al. Additional Figure 2: Sequence comparison of PCR primers for amplification of HML-2 *gag* (A) and *env* (B) cDNAs with primer binding regions in HML-2 proviral loci. Proviral loci were included when at least one primer binding region was present. HML-2 loci identified as transcriptionally active are listed first, followed by loci not identified as active in this study. Locations of the latter are given as chromosomal bands. Reverse primer sequences have been reverse-complemented. "yes" indicates that the corresponding HML-2 locus has been found transcriptionally active by detection of its *gag* and/or *env* cDNA. Note that locus c19_A consists only of *env*-LTR portions, and that loci c3_D and c10_B lack relevant *env*-LTR portions (see Additional Fig. 1).
